# Evaluating consistency of radiomic features derived from CT images: A cross‐center phantom study

**DOI:** 10.1002/acm2.70482

**Published:** 2026-01-31

**Authors:** Lorna Tu, Hervé H. F. Choi, Haley Clark, Bradford Gill, Scott Young, Samantha A. M. Lloyd

**Affiliations:** ^1^ Department of Physics and Astronomy University of British Columbia Vancouver British Columbia Canada; ^2^ Department of Medical Physics BC Cancer Vancouver British Columbia Canada; ^3^ Department of Medical Physics BC Cancer Surrey British Columbia Canada

**Keywords:** computed tomography, feature consistency, multi‐institutional, phantom, radiomics

## Abstract

**Purpose:**

To investigate the consistency of radiomic features extracted from computed tomography (CT) scans across CT radiotherapy simulators geographically spread across a Canadian province using a simplified lung radiomic phantom, and to determine whether it is appropriate to combine multicenter imaging data into a single dataset.

**Methods:**

An inexpensive phantom was created using foam with a density similar to lung and a plastic vial insert filled with water. It was imaged at six provincial radiotherapy treatment centers using eight GE CT radiotherapy simulators and routine lung stereotactic ablative radiotherapy planning CT acquisition protocols. Radiomic features were extracted from regions of interest using Imaging Biomarker Explorer radiomics software and compared using Kruskal Wallis H tests, intraclass correlation coefficient (ICC), and coefficient of variation (CV).

**Results:**

Image acquisition parameters were similar across centers. At the population level, no significant inconsistencies between radiomic features originating from different centers or from within the same center were observed (Bonferroni‐corrected *p *> 0.05; ICC > 0.941). On average, 52.5% of features were considered consistent (CV ≤ 0.10).

**Conclusions:**

The proposed phantom was transported across widespread centers without detectable damage and demonstrates potential for easy quality assurance checks on radiomic feature consistency within a multi‐institutional setting. Our analysis suggests that some features should be omitted or standardized before combining provincial imaging data into a harmonized lung radiotherapy dataset. These preliminary findings lay the groundwork for further investigation into provincial radiomic feature consistency and potential application to multicenter clinical studies. Owing to potential differences in imaging protocols, a consistency evaluation should be performed before undertaking radiomic analysis of data combined from different institutions.

## 1 INTRODUCTION

Radiomics involves extracting quantitative features from medical images, reducing the dimensionality, and narrowing in on salient aspects that may have clinical value. Patterns in radiomic features may be used to improve cancer diagnosis and prognosis, predict treatment response,^1^, ^2^ and potentially support clinical decision making as a personalized medicine tool.^3^, ^4^

Combining data in multicenter radiomic collaborations can potentially increase statistical power and more accurately represent the broader patient population. However, the use of different scanner manufacturers, image reconstruction algorithms, and acquisition protocols may add unwanted variability in the data.^5^ Radiomic features are generally sensitive to CT image reconstruction techniques^6^, ^7^ and acquisition parameters such as tube current and noise index.^8^ These factors may cause feature variations that interfere with robust radiomic analysis.

Feature consistency should be verified before multicenter images are combined for analysis. A set of multicenter phantom CT scans is publicly available but, to our knowledge, has not been used for benchmarking purposes.^9^ Instead, feature consistency is assumed due to the use of standardized image acquisition protocols in clinical trials,^10^ which is not possible for retrospective studies and not guaranteed if different scanners are used.^5^ As an alternative to standardizing the image acquisition stage, harmonization methods may be applied post‐acquisition.^11^ For example, ComBat^12^ was originally introduced to harmonize gene expression array data but has since been applied to harmonize radiomic features.^13^ ComBat adjusts the batch effect of data and requires a representative sample, which is a limitation in multicenter studies with a small number of patients per center. Despite the potential feature inconsistency across centers, harmonization is not consistently done in radiomic studies.^14^ Preliminary feature verification would evaluate whether data harmonization measures are required to standardize features of a particular dataset. Feature verification may involve scanning a benchmarking phantom at different centers as a form of quality assurance. The use of phantom data instead of patient data allows for more comprehensive feature assessment with no direct risk to patient safety. Since the same phantom is considered, a certain set of feature values is expected; any differences across institutions would be attributed to center‐specific factors. Standardized phantom benchmarking offers a direct comparison of features across institutions and a better picture of what post‐acquisition harmonization methods are required in subsequent multi‐center radiomics collaborations.

The purpose of this work is twofold. First, we propose a low‐cost, low‐complexity phantom which can be easily transported across centers and be used to compare radiomic feature consistency across multiple CT simulators and institutions on the basis of lung radiotherapy imaging protocols. Second, we evaluate the feasibility of using the phantom for quality assurance checks on radiomic features extracted from a single institution with multiple centers and heterogeneous CT scanner hardware and protocols as preparatory work for generating a large, multicenter dataset.

## 2 METHODS

### 2.1 Phantom development

Using computer‐aided design software (SolidWorks; Dassault Systèmes, Waltham, MA), a physical radiomic phantom was created with the following design goals: (1) include lung‐related materials of radiological interest (flexibility to tailor toward relevant tissues or features), (2) reproducible and low‐cost manufacturing, and (3) durable yet compact and lightweight to simplify transport and storage.

**FIGURE 1:**
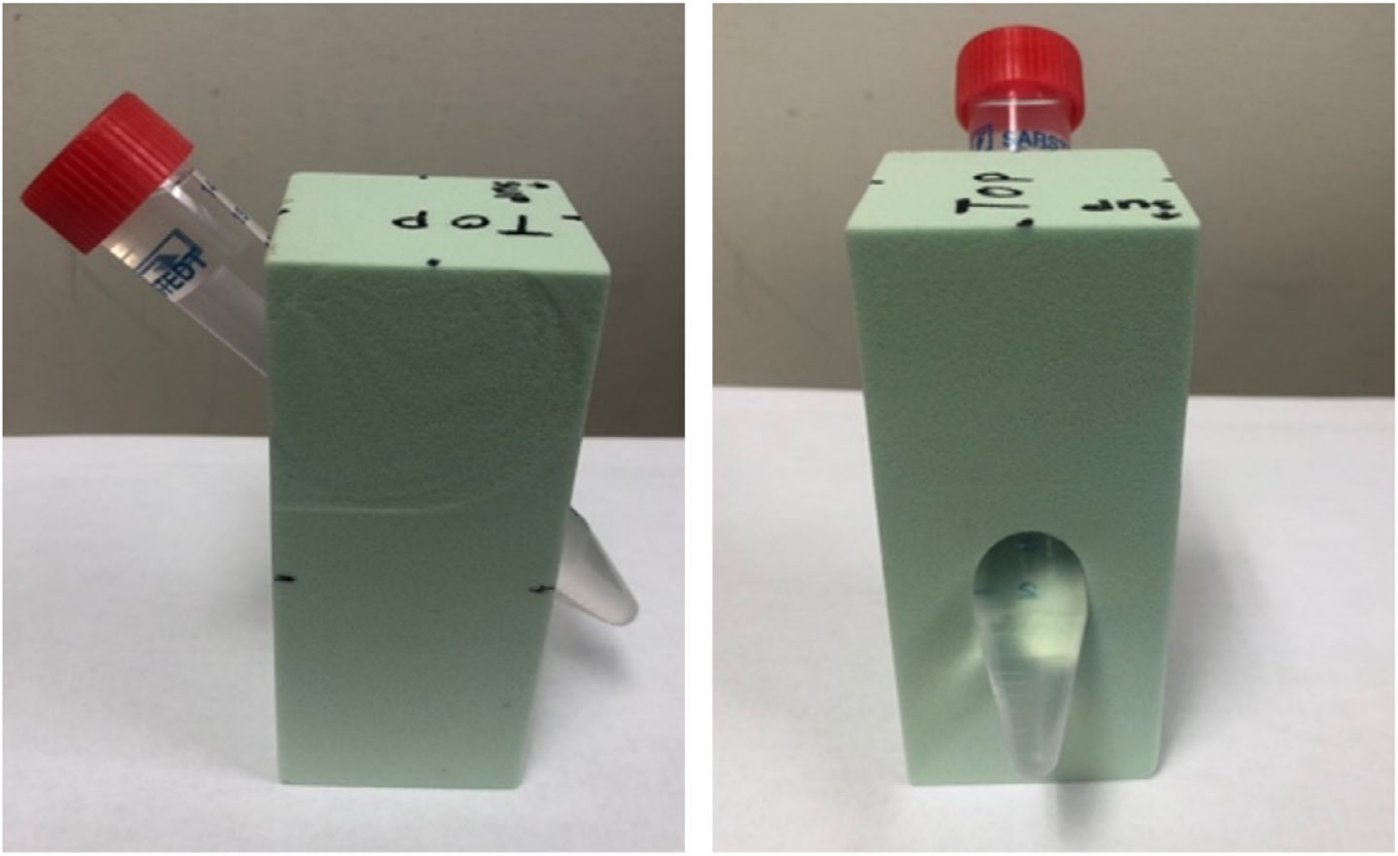
Radiomic phantom made of lung‐equivalent foam and plastic vial insert.

The phantom measures 10 × 4 × 4 cm^3^ (Figure 1). It was manufactured in‐house using foam (CORAMFOAM U150; Freeman Manufacturing & Supply Company, Avon, OH) with a lung‐equivalent density of 0.24 g/cm^3^, and includes an angled (45°) and tapered space to secure a 14 mL plastic vial insert. The angled space allows the insert to be easily added or removed to the foam body, but also held securely in place when necessary, such as during a scan. The geometry also ensures that the air bubble in the insert is located outside the foam body, removing it from all axial slices to be analyzed. The insert can be filled with a variety of liquids; to mimic lung tumor anatomy in this study, it was filled with water. To simulate patients scanned with intravenous contrast, another insert was filled with contrast medium (Omnipaque 300; GE Healthcare Ireland, Cork, Ireland) diluted to 14% with water. The phantom weighs less than 50 g with the water insert. Markings for consistent positioning via external laser alignment were drawn by hand onto the phantom.

### 2.2 CT scanning

The phantom was imaged using eight 7^th^ generation CT General Electric radiotherapy simulators located in radiation oncology departments across six cancer treatment centers in the province of British Columbia (BC Cancer Abbotsford, Kelowna, Prince George, Surrey, Vancouver, and Victoria). Images were acquired using center‐specific routine lung stereotactic ablative radiotherapy planning acquisition protocols and manufacturer‐suggested default internal image reconstruction methods. Some centers routinely use a 4DCT in their routine lung radiotherapy protocols to account for respiratory motion; 4DCT was used in our work to fully replicate these protocols even though the phantom does not move. For 4DCT protocols (as indicated in Table 2), a motion device to simulate respiratory motion was placed on the couch away from the phantom. The motion device was within view of the respiratory tracking cameras so that images could be binned and produce an averaged image set that was analyzed. Additional scans varying a single CT setting were acquired using one CT simulator.

After transport to distant centers, the phantom was allowed to rest for at least twenty‐four hours before scanning so any air bubbles created inside the vial during transit would dissipate. The water level was also verified against a permanent fill line.

To validate the structural integrity of the phantom after transport, it was scanned before and after transport to another center. Paired scans from an independent scanner were compared.

### 2.3 Radiomic feature extraction

**FIGURE 2:**
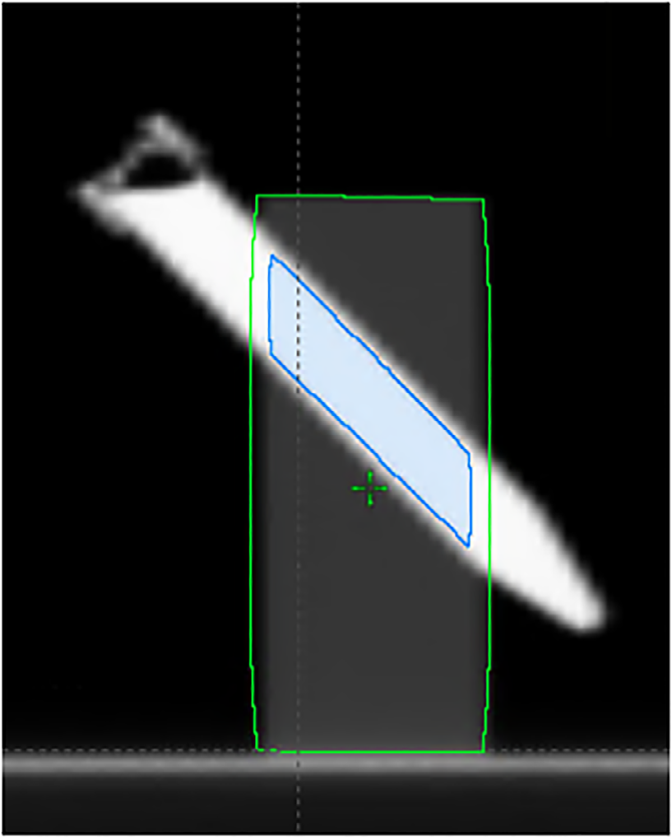
Regions of interest delineated on a CT scan of the phantom. The lung‐equivalent foam body and insert are outlined in green and blue, respectively.

Images were contoured in the Varian Eclipse Contouring 15.6 workspace by a single individual. The window/level was adjusted to the lung pre‐set and the Geometrical Shape and interpolation tools were used to contour the foam block. The insert was contoured using the brush tool. Image slices protruding beyond the foam block were excluded from the region of interest (ROI). The insert was cropped back one slice from either end of the foam block contour. The contours were then copied to subsequent registered scans to minimize contour variation and ensure consistency. The foam block and vial insert ROIs are shown in Figure 2. Sixty‐one radiomic features were extracted from each of the two ROIs (lung‐equivalent foam block and insert) using the IBEX radiomics software package.^15^ Features are listed in Table 1 and include five classes of radiomic features.

**TABLE 1:** Radiomic features used in this study.

Category	Feature name
GrayLevelCooccurenceMatrix25	AutoCorrelation
	ClusterProminence
	ClusterShade
	ClusterTendendcy
	Contrast
	Correlation
	DifferenceEntropy
	Dissimilarity
	Energy
	Entropy
	Homogeneity
	Homogeneity2
	InformationMeasureCorr1
	InformationMeasureCorr2
	InverseDiffMomentNorm
	InverseDiffNorm
	InverseVariance
	MaxProbability
	SumAverage
	SumEntropy
	SumVariance
	Variance
GrayLevelRunLengthMatrix25	GrayLevelNonuniformity
	HighGrayLevelRunEmpha
	LongRunEmphasis
	LongRunHighGrayLevelEmpha
	LongRunLowGrayLevelEmpha
	LowGrayLevelRunEmpha
	RunLengthNonuniformity
	RunPercentage
	ShortRunEmphasis
	ShortRunHighGrayLevelEmpha
	ShortRunLowGrayLevelEmpha
NeighborIntensityDifference25	Busyness
	Coarseness
	Complexity
	Contrast
	TextureStrength
IntensityDirect	EnergyNorm
	GlobalEntropy
	Kurtosis
	GlobalMean
	GlobalMedian
	Skewness
	GlobalStd
	GlobalUniformity
Shape	Compactness1
	Compactness2
	Convex
	ConvexHullVolume
	ConvexHullVolume3D
	Mass
	Max3DDiameter
	MeanBreadth
	Orientation
	Roundness
	SphericalDisproportion
	Sphericity
	SurfaceArea
	SurfaceAreaDensity
	Volume

### 2.4 Statistical analysis

Features were compared with Kruskal‐Wallis H tests in the Python scipy‐stats^16^ (version 1.7.3) library. The Kruskal‐Wallis H test is a nonparametric ANOVA test that tests whether two or more independent samples originate from the same distribution. It was used to compare two feature groups at a time: features between two simulators from different centers or features between two scans acquired with the same simulator (either from varying a single acquisition parameter or from scanning with an independent simulator to validate structural integrity).

The intraclass correlation coefficient (ICC) in the Python pingouin^17^ (version 0.5.5) library was used to compare features extracted from images acquired using a fixed set of simulators (using ICC3). ICC values less than 0.75 indicate poor to moderate reliability, while values between 0.75 and 0.90 indicate good reliability and values greater than 0.90 indicate excellent reliability.^18^ The median and interquartile range were also computed for each feature.

The coefficient of variation (CV) in the Python scipy‐stats^16^ (version 1.7.3) library was used to evaluate individual feature consistency. CV is defined as the standard deviation divided by the mean. A low CV would indicate that a feature's variability is small relative to its mean, suggesting greater stability. Since CV depends on the mean, features that contain both positive and negative values would result in a smaller mean, which artificially inflates the CV. The Shape feature, Orientation, originally contained values ranging from −90° to 90°; its values were shifted to range from 0° to 180° for the purposes of calculating CV. The absolute values of the CVs were used to categorize feature variation in three groups: low (CV ≤ 0.10), moderate (0.10 < CV ≤ 0.20), and high (CV > 0.20).

## 3 RESULTS

### 3.1 Phantom fabrication

The cost of raw materials was approximately $25 CAD per phantom. The design is sufficiently simple that it can be constructed in about two hours by a radiation therapy department machine shop equipped with foam‐cutting tools. The design files and fabrication details are available on GitHub (https://github.com/loronaut/RadPhant1).

### 3.2 Phantom transportation

The phantom was transported in a protective case. Eleven trips throughout the province were completed without any observable damage to the phantom or vial. It travelled a total of approximately 2723 km. The shortest trip was 34 km and the longest trip was 785  km.

The phantom was scanned using the same simulator before and after completing two trips throughout the province (a total distance of about 72 km). No visible damage or change was observed in the phantom and the scans. Statistical analysis did not show any differences between the scans (Bonferroni‐corrected *p *> 0.05 and ICC > 0.99).

### 3.3 Image acquisition parameters

Center‐specific image acquisition parameters for routine lung protocols are shown in Table 2 and include tube voltage, tube current, exposure time, exposure, and generator power. Parameters were similar, apart from a wide range of generator power (ranging from 4800 to 52800 kW) and tube current (ranging from 10 to 100 mA) due to variation in automatic exposure settings. Standard convolution kernel and 2.5 mm slice thickness were most common.

**TABLE 2:** Routine CT image acquisition parameters at provincial centers.

Center	Scanner model	Tube voltage (kV)	Tube current (mA)	Exposure time (s)	Generator power (kW)	Convolution kernel	Slice thickness (mm)
A1	LightSpeed RT16	120	150	1782	18000	Standard	2.5
A2^a^	Advantage 4D	120	10	800	52800	Lung	2.5
A3^a^ ^,b^	Discovery RT	120	99	800	52800	Standard	2.5
B	Optima CT580	120	20	500	48000	Standard	2.5
C^a^	Advantage 4D	120	10	800	52800	Standard	2.5
D	Optima CT580	120	10	800	52800	Standard	2.5
E	Optima CT580	120	100	1503	5280	Lung	2.5
F	LightSpeed RT16	120	40	4000	4800	Standard	1.25

^a^ indicates 4DCT protocol.

Additional nonroutine parameters for a Discovery RT simulator at one center are shown in Table 3 and include tube current, convolution kernel, slice thickness, and insert material that differ from the default protocol.

**TABLE 3:** Nonroutine CT image acquisition parameters for a Discovery hRT unit at one center (A3).

Protocol	Tube current (mA)	Exposure time (s)	Generator power (kW)	Convolution kernel	Slice thickness (mm)	Insert material
helical	225	11608	27000	Detail	2.5	Water
axial	225	800	27000	Detail	2.5	Water
lung	100	800	12000	Lung	2.5	Water
FOV50cm	100	800	12000	Standard	2.5	Water
default	100	800	12000	Standard	2.5	Water
contrast	100	800	12000	Standard	2.5	Contrast
150mA	150	800	18000	Standard	2.5	Water
10mA	10	800	1200	Standard	2.5	Water
5mm	100	800	12000	Standard	5	Water
3.75mm	100	800	12000	Standard	3.75	Water
1.25mm	100	800	12000	Standard	1.25	Water

### 3.4 First‐order statistics

**FIGURE 3:**
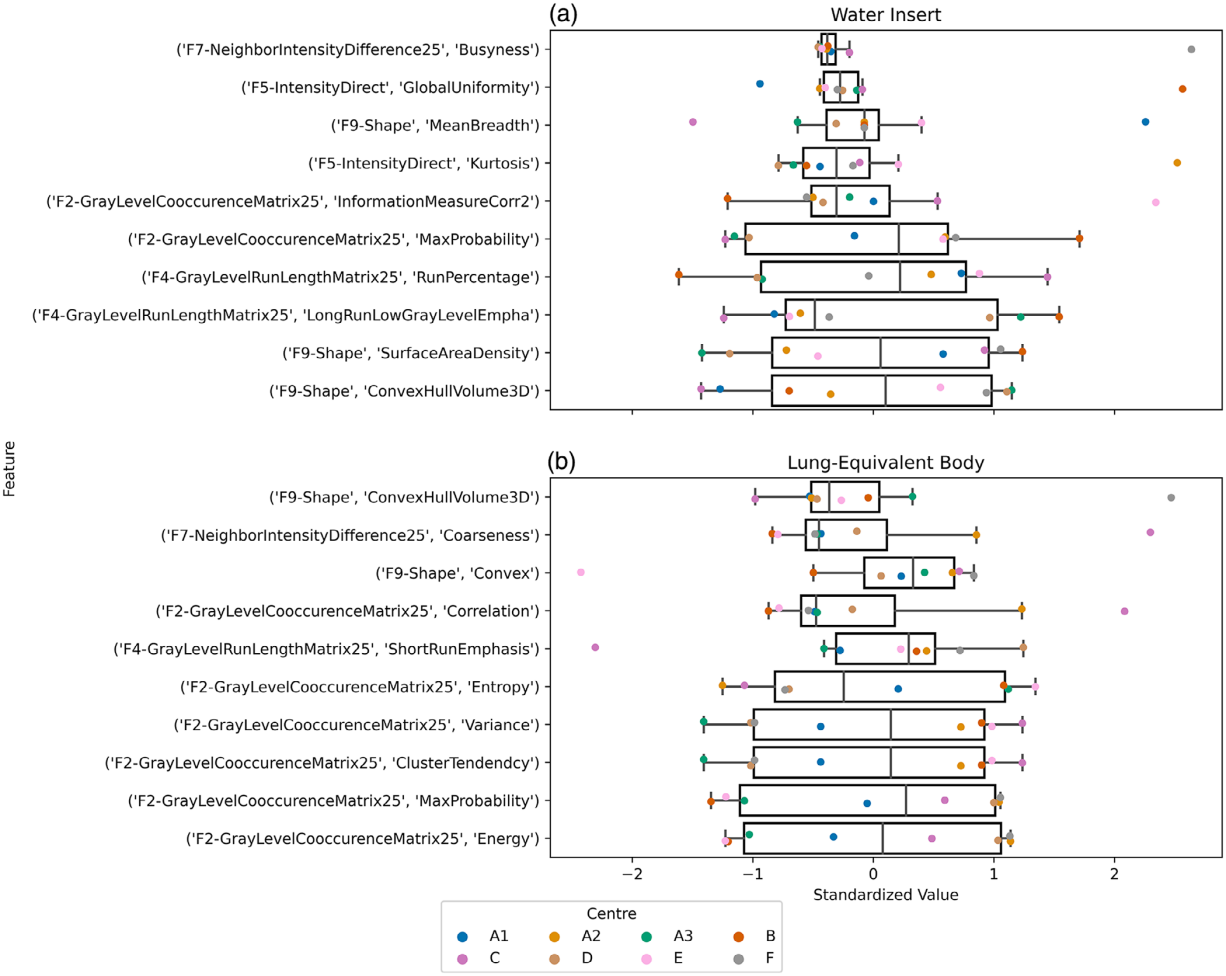
Boxplots of the five most and least reproducible features across eight scanners based on interquartile range for (a) water insert ROI and (b) body ROI. Feature values were standard scaled for display. The most reproducible features are the uppermost five boxplots and the least reproducible are the lower five boxplots of each subplot.

The medians ranged from the order of 10^−1^ to 10^6^. Due to the wide range of values, feature distributions were standard scaled to have a mean of 0 and a standard deviation of 1 before determining their interquartile range. The five most and least reproducible features based on interquartile range are presented as boxplots in Figure 3. Without standardization, the least reproducible features ranged from 0.11 to 0.33 for the body ROI, and 6.57 to 7.70 for the water ROI.

### 3.5 Population‐level center comparison

**FIGURE 4:**
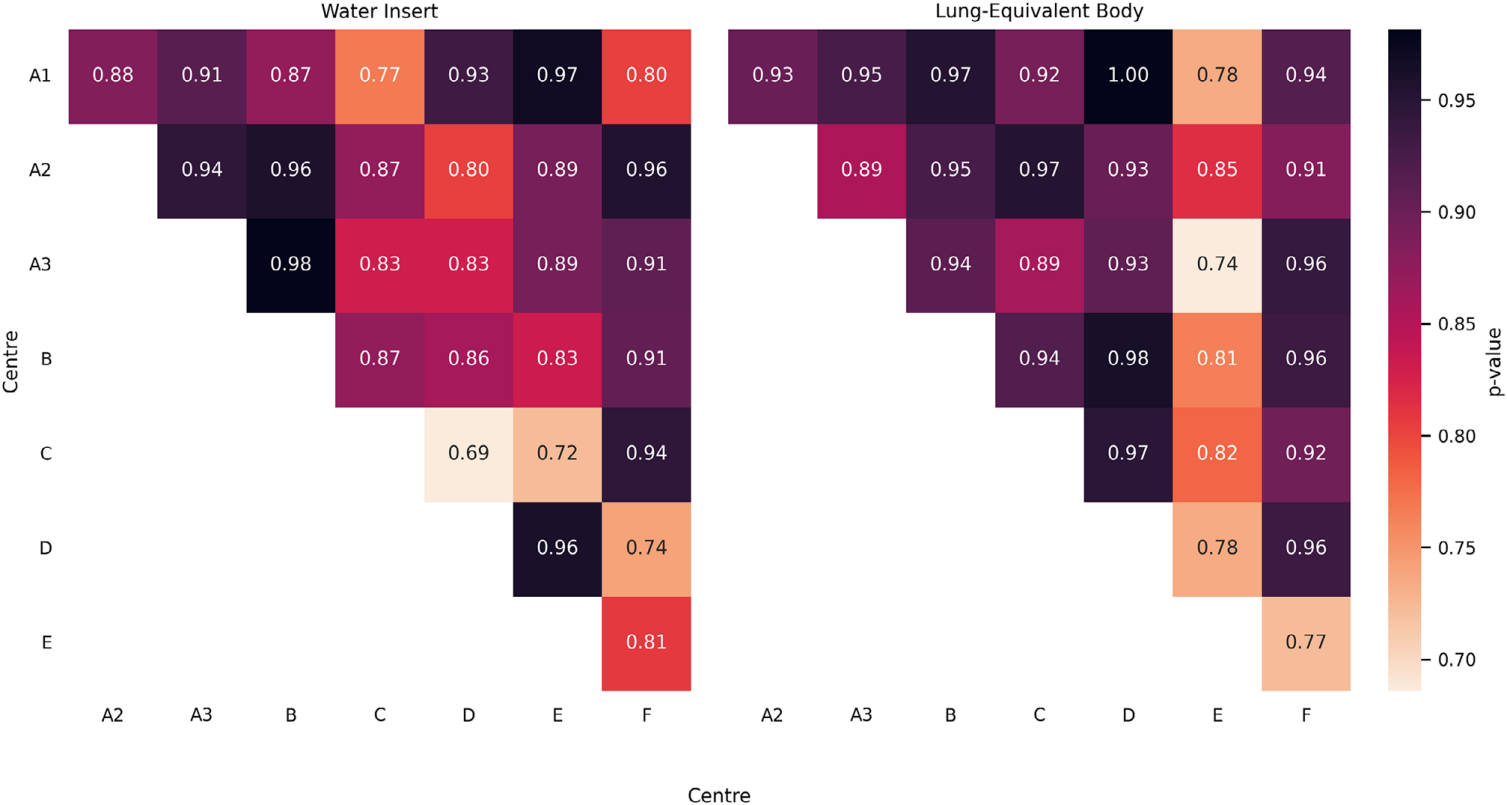
*p*‐values from Kruskal‐Wallis comparisons between scanners at provincial centers for two regions of interest involving water and lung‐equivalent material. No significant difference between scanners was observed (Bonferroni‐corrected *p *> 0.05).

Figure 4 shows heatmaps of feature comparison *p*‐values for scans from each center. Variations may be attributed to differences in image acquisition parameters and are especially prominent if a lung convolution kernel is applied to a water region or vice versa. At the population level, no statistically significant difference in radiomic features between centers was observed (all Bonferroni‐corrected *p *> 0.05, with *p *= 0.69 as the lowest). ICCs for water and body ROIs were 0.998 and 0.957, respectively, which indicates excellent reliability. At the population level, no significant differences were observed when comparing three simulators within the same center (ICCs were 0.997 and 0.941 for water and body ROIs, respectively).

**FIGURE 5:**
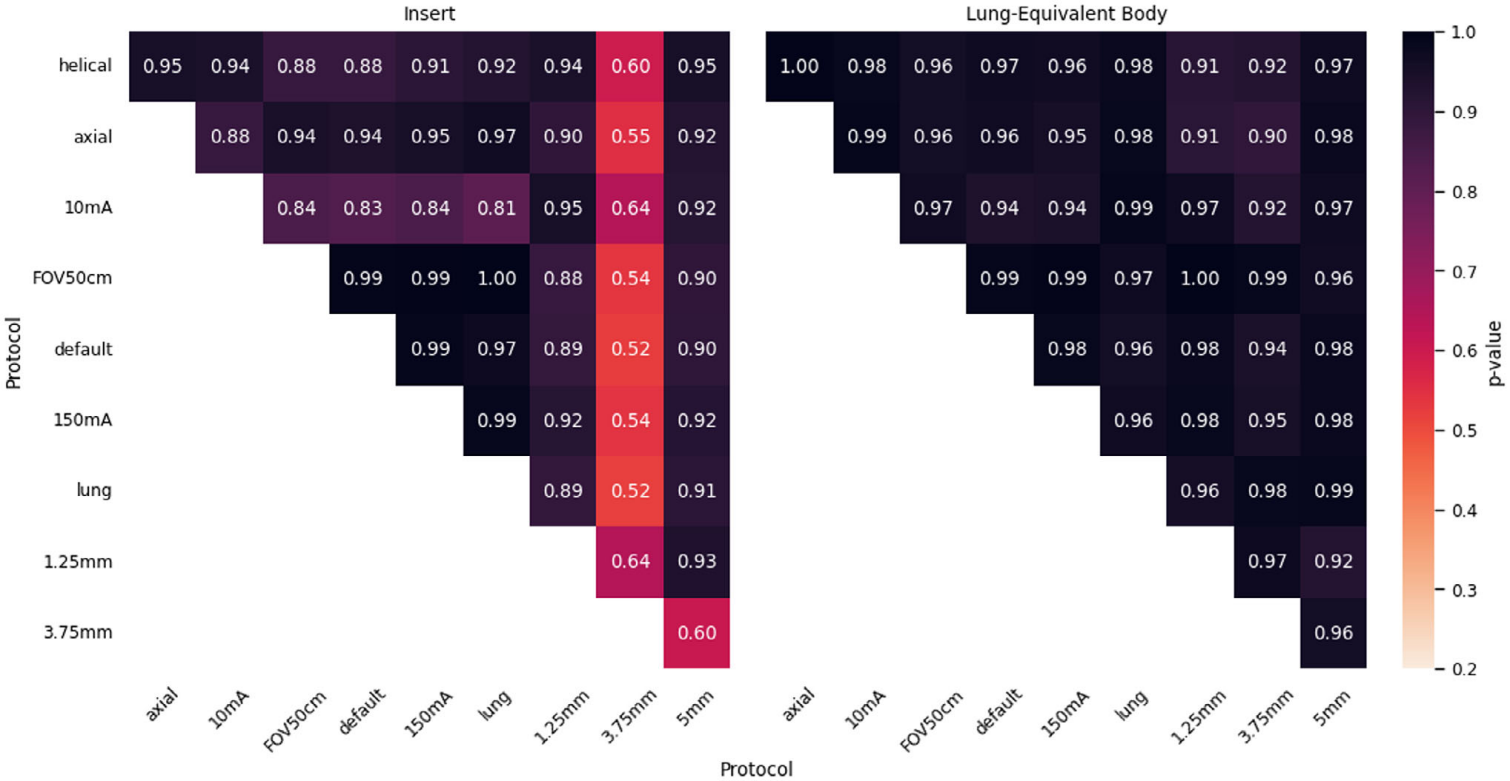
*p*‐values from Kruskal‐Wallis comparisons for images acquired using the same scanner, varying one setting from the routine default protocol. The regions of interest are the plastic vial with a water insert and lung‐equivalent body. No significant difference between protocols was observed (Bonferroni‐corrected *p *> 0.05).

Similarly, Figure 5 shows heatmaps of *p*‐values when the default protocol was altered by a single acquisition parameter. Varying one parameter at a time resulted in some observable differences, although all were far from reaching the threshold for statistical significance. In comparing the *p*‐values for each feature category, shown in Supplementary Figure S1 heatmaps, a difference was observed when images were acquired using a 3.75 mm slice thickness but not other thicknesses. The use of a 10 mA tube current or the lung reconstruction kernel respectively did not have a statistically significant effect on shape or texture features for the water insert ROI (with *p *= 0.35 as the lowest). The use of a contrast insert instead of water also resulted in an observable but not statistically significant difference (with *p = *0.27 as the lowest).

### 3.6 Individual feature consistency

CVs for each ROI are reported in Supplementary Table S1. They ranged from 1 × 10^−4^ to 2.193 for the water ROI, and 0.002 to 0.858 for the body ROI. On average, 52.5%, 13.9%, and 33.6% of features extracted from water and body ROIs had low, moderate, and high CVs respectively. The most consistent features for both ROIs were InverseDiffMomentNorm, InverseDiffNorm, and Convex (from the GrayLevelCooccurenceMatrix25 and Shape feature categories). The least consistent features were Busyness, Complexity, and Correlation (from the NeighborIntensityDifference25 and GrayLevelCooccurenceMatrix25 categories) for the water ROI, and GrayLevelNonuniformity, LongRunEmphasis, and LongRunHighGrayLevelEmpha (from GrayLevelRunLengthMatrix25) for the body ROI.

## 4 DISCUSSION

This preliminary study was conducted to (1) propose a simplified radiomic phantom suitable for cross‐center transport, and (2) evaluate its feasibility for quality assurance checks of radiomic feature consistency across a single institution with multiple distributed centers.

The design of the phantom was intentionally kept as simple as possible with the idea that it might need to be reconstructed on‐site for each scan. The low cost and low‐complexity design increases manufacturing options and consistency, including making a phantom on‐site for centers equipped with fabrication facilities; fabrication of several identical phantoms in a centralized facility for distribution to other centers; and fabrication of a single phantom that can be shipped between centers one‐at‐a‐time. To gauge the durability of our design, we opted for the last option. After being transported eleven times for a total distance of approximately 2723 km, the phantom incurred no observable damage, suggesting the design and materials are suitable for wide distribution. We recommend encasing the phantom in a protective outer case during shipping. The low total cost of fabrication makes all aforementioned distribution models viable, including scenarios where shipping between centers is unreliable (e.g., one time use).

The results of distributional comparisons did not demonstrate statistically significant changes between radiomic features originating from differences in local imaging equipment and protocols, indicating population‐level similarity. Although slice thickness and reconstruction method were previously found to have the most potential to affect features,^7^ these parameters were similar or identical across centers in our study. Slight differences seen in the heatmaps of Figure 4 may be attributed to the use of GE's standard reconstruction kernel which creates smoother and less noisy images, as opposed to the lung reconstruction kernel.^3^, ^19^ In our study, the use of the lung construction kernel affected some features as expected, such as texture features from the GrayLevelRunLengthMatrix25 and NeighborIntensityDifference25 categories.

Our analysis suggests that some features are more stable than others. Although the boxplots in Figure 3 show that the top five stable features have small interquartile ranges, the presence of outliers suggests potential instability. While boxplots were used to summarize distributional characteristics, CVs were used to quantitatively assess feature consistency.

In general, first‐order and shape features are more consistent than texture features,^7^, ^20^ which aligns with our analysis. All Shape features except for Orientation had low CVs and were considered consistent. Since Orientation depends on the angle, it may be sensitive to small changes in positioning and contouring. While GrayLevelRunLengthMatrix25 texture features were the least consistent, texture features in the GrayLevelCooccurenceMatrix25 category were among the most and least stable. Prior work using a homogeneous water phantom determined that texture features were sensitive to slice thickness, but not tube voltage or tube current.^20^, ^21^ Although a significant effect of slice thickness was not observed in our study at the population level (Figure 5), CVs indicate that it may have affected the stability of individual texture features. While thicknesses of 1.25 and 2.5 mm are routinely used for clinical lung radiotherapy CT scans in our centers, we also investigated the effect of thicknesses up to 5 mm. Thinner slice images contain smaller partial volume artifacts and less smoothing compared to a thicker technique,^19^ which would affect texture feature computation. A slice thickness of 3.75 mm resulted in an observable change in texture and shape features while 5 mm did not. This is likely due to the finite number of CT detectors and their physical width compared to the desired slice thickness; 1.25 mm, 2.5 mm, and 5 mm are integer multiples of the detector width whereas 3.75 mm is not. In our study, we also found that texture features are not affected by changing the tube current. However, the use of a 10 mA tube current caused an observable but not significant effect on shape features, likely due to boundary shift. In addition, the identical tube voltage, similar tube current, and lack of heterogeneity in the phantom's materials may not have contributed any detectable feature variation.

This study has several limitations. First, it used a nonanatomic phantom made of two low density, homogeneous materials. Considering only two materials limits the generalizability of the results, as the features would be most applicable to water‐ and lung‐equivalent structures. Since only low density materials were included, the impact of density on radiomics uncertainty and sensitivity to acquisition parameters was not explored. Analysis of individual feature reproducibility shown in the boxplots of Figure 3 and CVs suggests that some features may be more reproducible depending on the material. Other features have low CVs for both water and body ROIs, so their reproducibility may be independent of density and potentially more generalizable. Materials with more varied texture or higher attenuation coefficients, such as bone, are not represented in the proof‐of‐concept phantom and will be investigated in subsequent work. Although diluted contrast medium was used in this study, it did not result in a statistically significant effect on radiomic features in that region of interest. This may be because the low iodine concentration is not detectable by our analysis. Future iterations of the phantom may include a series of contrast concentration to determine the minimum concentration required for detection or other low‐cost, common materials of radiological interest for other non‐lung applications, such as mineral oil, alcohol, or sand, in the plastic vial insert. Textured materials are of particular interest. Furthermore, it is unclear to what extent the results can be translated to CT scans of human subjects.

Second, the study did not explore the full range of all CT acquisition parameters. While images using the full range of slice thicknesses were acquired, other parameters were not considered as extensively. Although it may be less informative, the use of parameters that are currently used in routine clinical practice are more relevant and applicable for radiomic studies at our institution. In particular, a harmonized approach to stereotactic radiotherapy planning and treatment provincially was assumed to be advantageous in the setting of radiomic data extraction, and to our knowledge this is the first time this assumption has been explicitly tested.

Third, radiomic features were calculated using only one radiomics software package. While IBEX is a widely used software package, the use of another package could conceivably lead to different results due to ongoing challenges with feature reproducibility. Features extracted by other software, such as slicer or ITK‐snap, could be used for horizontal or vertical comparison in future work. In particular, other software would enable the calculation of wavelet features, which are not available in IBEX, for more comprehensive analysis. Future studies may also include software that is compliant with the Image Biomarker Standardisation Initiative (IBSI),^22^ though compliance would still not guarantee feature reproducibility.^23^, ^24^

Last, stable features were primarily identified based on one quantitative reproducibility metric, CV. There is no consensus for the choice of cut‐off value used to identify features with low variation, yet it affects the results. A rigorous threshold of 0.10 was chosen because the study involves scanning a single phantom, which is expected to introduce less variability than a patient. While CV ≤ 0.10 identifies consistent features, it may disregard useful features that are close to the threshold, such as Entropy (0.106 and 0.120 for water and body ROIs respectively), and have been reported as stable in previous studies.^6^ Due to its dependence on the mean, CV may produce spurious results when the mean is close to zero or when the data is not normally distributed. Since this study's data originates from scanning the same phantom, a normal distribution is very likely but cannot be fully verified with eight scans. Future studies will involve a deeper test‐retest investigation featuring additional scans of multiple phantoms scanned across different CT simulators. In addition, multiple scans would enable ICC calculations for each individual feature. A consensus between ICC and CV values may more conclusively identify consistent features.

Our study found that a subset of features may be consistent, but does not recommend a definitive course of action for treating inconsistent features before further radiomic study. Features with low CV would suggest greater stability and would be most suitable for multi‐center radiomic collaborations. Features with moderate CV should be used carefully, and features with high CV should likely be omitted from the study or undergo post‐scan harmonization that will be explored in future work. While shape features are more stable, higher‐order texture features capture underlying information in medical images that may hold exceptional clinical value.

## 5 CONCLUSION

Our phantom can be transported without damage between geographically widespread radiation therapy departments and be used as an easy quality assurance check within a multi‐institutional setting to validate radiomic features derived from CT images collected using routine lung stereotactic radiotherapy protocols. Our analysis of two density regions of interest found 52.5% of features were consistent across multiple centers within the same institution, suggesting that post‐scan harmonization or omission of unstable features may be required in the context of lung. Both the scanning protocol and radiomic features of interest should be carefully considered when creating multi‐center integrated datasets for robust analysis. The observed feature consistency implies that robust multi‐institutional radiomic analysis may be achieved under the same conditions, including scanners, acquisition parameters, and material densities. Care must be taken to investigate the variability of features before combining data from a different set of institutions.

## AUTHOR CONTRIBUTIONS

The study was designed by Lorna Tu, Hervé HF Choi, Haley Clark, and Samantha AM Lloyd. The phantom was created by Hervé HF Choi, Bradford Gill, and Scott Young. Data analysis was performed by Lorna Tu. The manuscript was drafted by Lorna Tu, and critical revisions were made by all authors.

## CONFLICT OF INTEREST STATEMENT

The authors declare no conflicts of interest.

## Supporting information



Supporting Information

## Data Availability

The data that support the findings of this study are available from the corresponding author upon reasonable request. The phantom design and fabrication instructions are available on GitHub (https://github.com/loronaut/RadPhant1).
