# Supplementary material for: Evaluating consistency of radiomic features derived from CT images: A cross‐center phantom study
Source: J Appl Clin Med Phys. 2026 Jan 31;27(2):e70482. doi: 10.1002/acm2.70482 (PMC12860509; doi:10.1002/acm2.70482)
Supplement: Supplementary file 1 — Supporting Information [file ACM2-27-e70482-s001.docx]

**SUPPLEMENTARY MATERIAL**

**Evaluating consistency of radiomic features derived from CT images: a cross-centre phantom study**

Supplementary Fig. S1: p-values from Kruskal-Wallis comparisons for images acquired using the same scanner, separated by feature category (a) GrayLevelCooccurenceMatrix25, (b) GrayLevelRunLengthMatrix25, (c) NeighborIntensityDifference25, (d) IntensityDirect, (e) Shape.

**(a)**


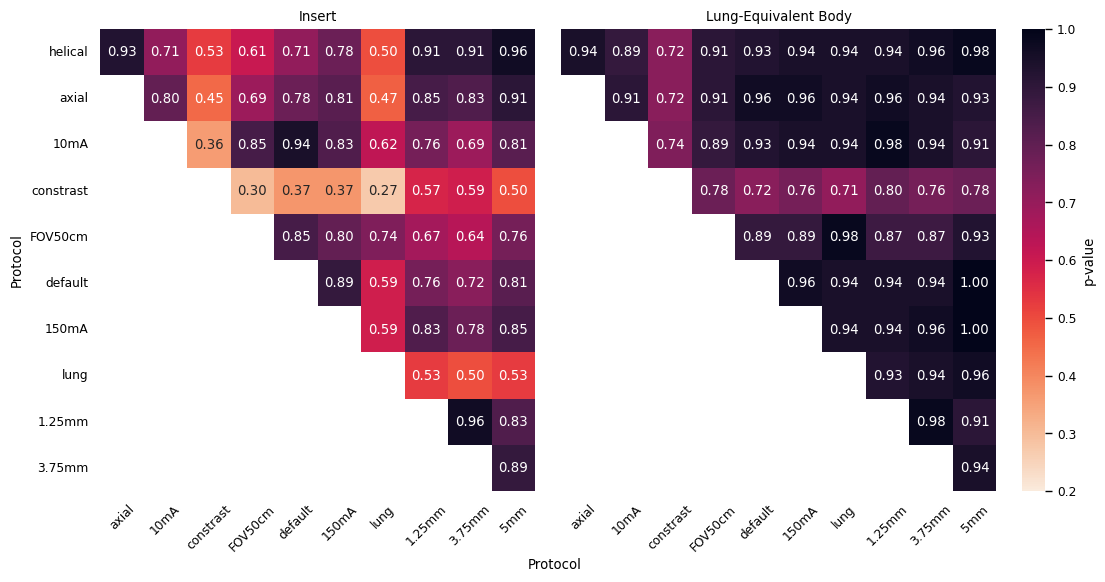


**(b)**


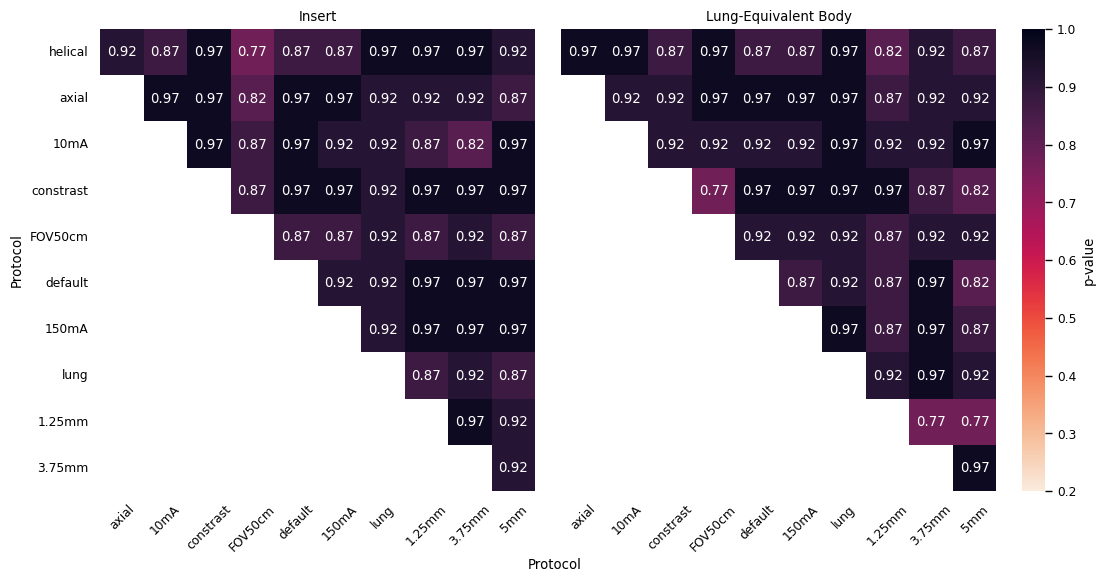


**(c)**


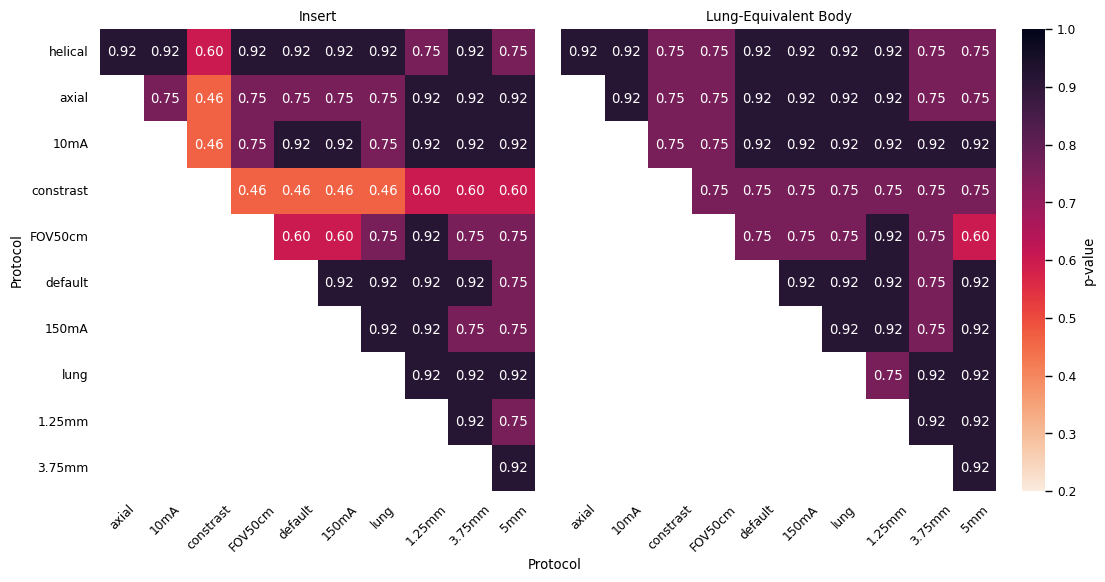


**(d)**


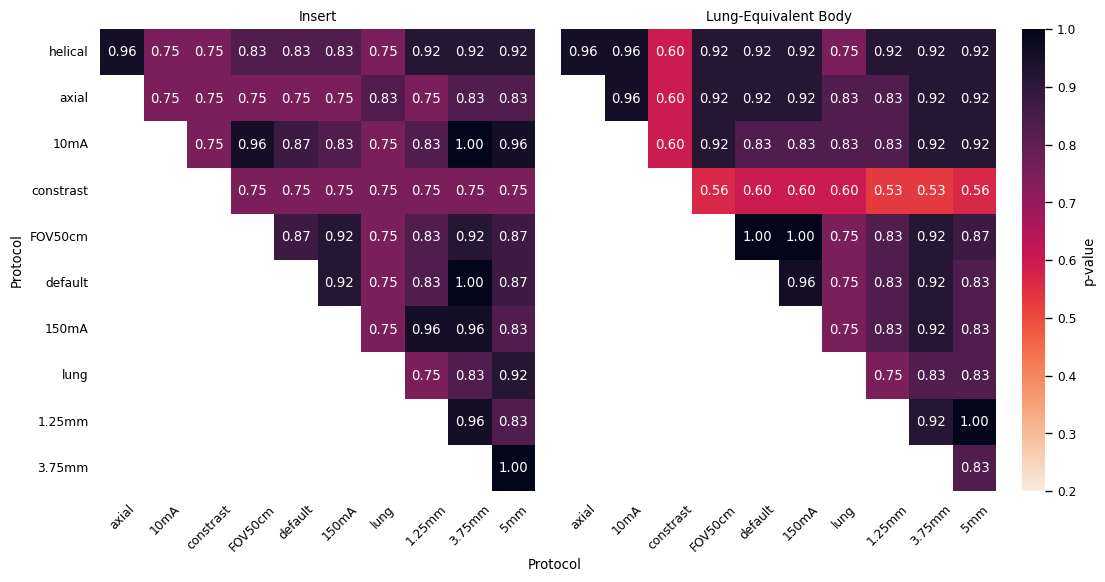


**(e)**


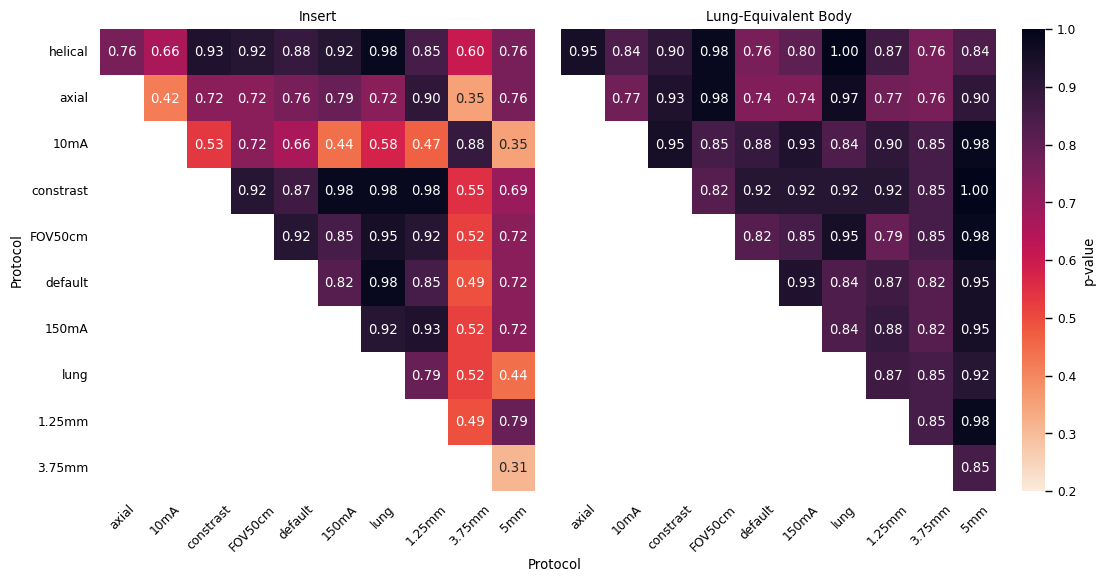


Supplementary Table S1: Coefficients of variation (CV) of each feature for water and body regions of interest.

| **Category** | **Feature Name** | **Water CVs** | **Body CVs** |
| --- | --- | --- | --- |
| GrayLevelCooccurenceMatrix25 | AutoCorrelation | 0.037 | 0.035 |
|  | ClusterProminence | 0.907 | 0.138 |
|  | ClusterShade | 0.682 | 0.120 |
|  | ClusterTendendcy | 0.325 | 0.053 |
|  | Contrast | 0.416 | 0.402 |
|  | Correlation | 0.921 | 0.169 |
|  | DifferenceEntropy | 0.070 | 0.158 |
|  | Dissimilarity | 0.133 | 0.318 |
|  | Energy | 0.331 | 0.448 |
|  | Entropy | 0.106 | 0.120 |
|  | Homogeneity | 0.035 | 0.083 |
|  | Homogeneity2 | 0.037 | 0.087 |
|  | InformationMeasureCorr1 | 0.184 | 0.182 |
|  | InformationMeasureCorr2 | 0.059 | 0.023 |
|  | InverseDiffMomentNorm | 0.000 | 0.002 |
|  | InverseDiffNorm | 0.001 | 0.006 |
|  | InverseVariance | 0.186 | 0.422 |
|  | MaxProbability | 0.265 | 0.383 |
|  | SumAverage | 0.018 | 0.019 |
|  | SumEntropy | 0.090 | 0.093 |
|  | SumVariance | 0.040 | 0.031 |
|  | Variance | 0.325 | 0.053 |
| GrayLevelRunLengthMatrix25 | GrayLevelNonuniformity | 0.241 | 0.858 |
|  | HighGrayLevelRunEmpha | 0.049 | 0.207 |
|  | LongRunEmphasis | 0.397 | 0.850 |
|  | LongRunHighGrayLevelEmpha | 0.395 | 0.792 |
|  | LongRunLowGrayLevelEmpha | 0.400 | 0.764 |
|  | LowGrayLevelRunEmpha | 0.045 | 0.282 |
|  | RunLengthNonuniformity | 0.198 | 0.456 |
|  | RunPercentage | 0.137 | 0.423 |
|  | ShortRunEmphasis | 0.076 | 0.089 |
|  | ShortRunHighGrayLevelEmpha | 0.115 | 0.221 |
|  | ShortRunLowGrayLevelEmpha | 0.075 | 0.245 |
| NeighborIntensityDifference25 | Busyness | 2.193 | 0.413 |
|  | Coarseness | 0.255 | 0.701 |
|  | Complexity | 0.985 | 0.588 |
|  | Contrast | 0.751 | 0.442 |
|  | TextureStrength | 0.848 | 0.320 |
| IntensityDirect | EnergyNorm | 0.036 | 0.038 |
|  | GlobalEntropy | 0.072 | 0.051 |
|  | Kurtosis | 0.869 | 0.008 |
|  | GlobalMean | 0.018 | 0.015 |
|  | GlobalMedian | 0.014 | 0.013 |
|  | Skewness | 0.298 | 0.013 |
|  | GlobalStd | 0.171 | 0.027 |
|  | GlobalUniformity | 0.161 | 0.195 |
| Shape | Compactness1 | 0.081 | 0.011 |
|  | Compactness2 | 0.159 | 0.039 |
|  | Convex | 0.012 | 0.002 |
|  | ConvexHullVolume | 0.073 | 0.007 |
|  | ConvexHullVolume3D | 0.065 | 0.014 |
|  | Mass | 0.062 | 0.018 |
|  | Max3DDiameter | 0.014 | 0.005 |
|  | MeanBreadth | 0.026 | 0.009 |
|  | Orientation | 0.708 | 0.249 |
|  | Roundness | 0.046 | 0.020 |
|  | SphericalDisproportion | 0.05 | 0.013 |
|  | Sphericity | 0.052 | 0.013 |
|  | SurfaceArea | 0.031 | 0.011 |
|  | SurfaceAreaDensity | 0.041 | 0.007 |
|  | Volume | 0.088 | 0.008 |
